# The association of remnant cholesterol inflammatory index with the risk of major adverse cardiovascular events in patients with angina undergoing percutaneous coronary intervention: a retrospective study

**DOI:** 10.3389/fcvm.2026.1837450

**Published:** 2026-06-17

**Authors:** Yazhao Sun, Xiao Yu, Chunlan Bai, Dongsheng Liu, Wenrui Xiong

**Affiliations:** 1 Department of Cardiology, Cangzhou People's Hospital, Cangzhou, Hebei, China; 2 Department of Neurology Intervention, Cangzhou People's Hospital, Cangzhou, Hebei, China; 3 Department of Scientific Research, Cangzhou People's Hospital, Cangzhou, Hebei, China

**Keywords:** coronary artery disease, high-sensitivity c-reactive protein, major adverse cardiovascular events, percutaneous coronary intervention, remnant cholesterol-inflammation index

## Abstract

**Background:**

The remnant cholesterol-inflammation index (RCII), which combines remnant cholesterol (RC) and high-sensitivity C-reactive protein (hs-CRP), reflects both metabolic and inflammatory risks. This study aims to evaluate the association between RCII and the risk of major adverse cardiovascular events (MACE) in patients with angina undergoing percutaneous coronary intervention (PCI).

**Methods:**

The association between baseline RCII and MACE risk was evaluated using multivariable Cox regression, restricted cubic spline (RCS) analysis, and time-dependent receiver operating characteristic (ROC) curves.

**Results:**

A total of 2,171 angina patients undergoing PCI were included (mean age 65.32 ± 6.85 years, 61.4% male), with a median follow-up of 36 months, during which 363 MACE events (16.7%) occurred. In the fully adjusted Cox model, each standard deviation increase in RCII was associated with a 5% higher MACE risk (HR = 1.05, 95%CI: 1.04–1.07); compared with the lowest tertile, the HRs for T2 and T3 were 2.87 (95%CI: 2.03–4.04) and 4.42 (95%CI: 3.09–6.32). Subgroup analysis revealed that RCII was significantly associated with MACE risk across different age groups (<65 years and ≥65 years), sex, smoking status, and the presence of coronary artery disease (CAD) history, diabetes, and hypertension, with no significant interactions observed between subgroups. RCS analysis revealed a non-linear positive association (*P* for non-linearity < 0.001). Time-dependent ROC analysis showed moderate discriminatory ability, with AUCs of 0.786 (12 months), 0.769 (18 months), 0.741 (24 months), and 0.739 (30 months).

**Conclusion:**

Baseline RCII may be a potential clinical biomarker for assessing the risk of new-onset MACE in patients with angina undergoing PCI.

## Introduction

Coronary artery disease (CAD) is a leading cause of death and long-term disability worldwide, posing a significant threat to patients' quality of life and overall health (1). As the population ages and risk factors persist, the burden of CAD remains high in many regions around the globe (2). Notably, there are substantial differences in this burden across countries with varying levels of socioeconomic development, suggesting significant opportunities for public health interventions (3). Angina, a common clinical manifestation of CAD, is primarily characterized by episodes of chest pain due to inadequate coronary blood supply. This condition profoundly impacts patients' quality of life and can lead to serious complications, including myocardial infarction. Despite considerable advancements in CAD treatment, particularly with the widespread use of percutaneous coronary intervention (PCI), many patients continue to face residual cardiovascular risk post-treatment, reflected in an elevated risk of major adverse cardiovascular events (MACE). A comprehensive registry study from Sweden, involving more than 100,000 post-myocardial infarction patients, revealed that the MACE risk during the first 365 days after myocardial infarction was 18.3%. Moreover, this risk gradually increased over time, with approximately 20% of patients experiencing new MACE within the following three years (4). Consequently, even after PCI, patients remain at substantial risk for cardiovascular events.

In recent years, lowering low-density lipoprotein cholesterol (LDL-C) levels has become the cornerstone of cardiovascular disease management. The 2019 ESC/EAS guidelines explicitly recommend (5) that, in individuals at very high cardiovascular risk, LDL-C should not only be reduced to <1.4 mmol/L but also decreased by at least 50%. Despite achieving these LDL-C targets, many patients continue to face a heightened risk of cardiovascular events, indicating the potential presence of other unrecognized residual cardiovascular risk factors. Recent studies have highlighted remnant cholesterol (RC) as an important independent risk factor for atherosclerosis. RC is primarily found in triglyceride-rich lipoprotein remnants, such as very low-density lipoprotein (VLDL), intermediate-density lipoprotein (IDL), and chylomicron remnants. When these remnants accumulate in the vasculature, they can penetrate the arterial endothelium, leading to endothelial dysfunction and inflammation, thus accelerating the formation and progression of atherosclerotic plaques (6). In addition to accumulating in the arterial walls, RC further contributes to plaque instability by promoting inflammation, increasing oxidative stress, and inducing the accumulation of immune cells (e.g., macrophages) within the plaques (7). A large-scale prospective study by Jung et al. found that elevated RC levels were significantly associated with an increased risk of MACE, an association that persisted across all age groups and remained independent of other traditional risk factors, including LDL-C levels (8). Furthermore, research by Esmaeili et al. demonstrated that RC is a stronger predictor of cardiovascular disease incidence than LDL-C (9).

Atherosclerosis is the central pathological process in cardiovascular disease, with dyslipidemia and vascular inflammation recognized as key drivers of its progression. Systemic low-grade inflammation has been identified as an important factor in the progression and instability of cardiovascular disease (10). Low-grade inflammation refers to a chronic, mild inflammatory state often characterized by subtle but persistent elevations in biomarkers such as high-sensitivity C-reactive protein (hs-CRP). Hs-CRP, as a biomarker of low-grade inflammation, has been confirmed as an independent predictor of cardiovascular events in numerous prospective cohort studies and meta-analyses (11, 12). Markus et al. found that elevated hs-CRP levels were significantly associated with the risk of future MACE, even after adjusting for traditional risk factors (13). Hs-CRP is an acute-phase reactant in the downstream inflammatory cascade, and its elevation reflects a systemic low-grade inflammatory state mediated by cytokines such as interleukin-6 (IL-6). These cytokines promote atherogenesis by enhancing pro-coagulant activity, increasing the adhesion of monocytes and leukocytes to endothelial cells, and stimulating the growth of vascular smooth muscle cells (14). A large prospective population cohort study demonstrated that even with long-term low LDL-C levels, persistently elevated hs-CRP significantly increased the risk of MACE (15). In our study, low-grade inflammation was assessed by measuring hs-CRP levels in the patients at baseline, helping to identify participants with a persistent low-grade inflammatory state, which could influence their long-term cardiovascular outcomes.

Based on the combined evaluation of RC and hs-CRP, we proposed the remnant cholesterol-inflammation index (RCII), a novel biomarker designed to simultaneously reflect the impact of dyslipidemia and chronic low-grade inflammation on cardiovascular risk. However, the relationship between RCII and the MACE risk in patients with angina who have undergone PCI remains insufficiently studied. This study aims to analyze data from PCI-treated angina patients to explore the association between baseline RCII levels and MACE risk, and to reveal how the combined exposure to RC and low-grade inflammation affects long-term prognosis. We hypothesize that RCII could provide a more accurate prognostic assessment, enhancing risk management in angina patients.

## Methods

### Study population

**Figure 1:**
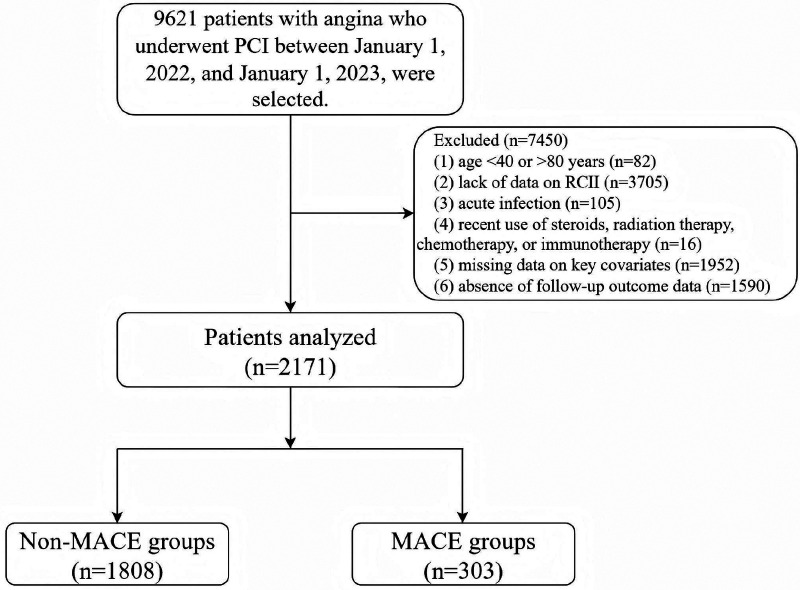
Flow diagram of patient selection. PCI*,* percutaneous coronary intervention; MACE, major adverse cardiovascular events.

This study is a single-center retrospective analysis conducted at Cangzhou People's Hospital (Cangzhou, Hebei, China) that included 9,621 consecutive patients with angina who underwent PCI between January 1, 2022, and January 1, 2023. Patients were sequentially excluded based on the following criteria: (1) age <40 or >80 years (*n* = 82); (2) lack of data on RCII (*n* = 3,705); (3) acute infection (*n* = 105); (4) recent use of steroids, radiation therapy, chemotherapy, or immunotherapy (*n* = 16); (5) missing data on key covariates (*n* = 1,952); (6) absence of follow-up outcome data (*n* = 1,590). Ultimately, 2,171 patients were included in the analysis (Figure 1).

### Ethics statement

This retrospective study was conducted in accordance with the Declaration of Helsinki and was approved by the Ethics Committee of Cangzhou People's Hospital (K2025-130-02), and all participants signed informed consent forms.

### Covariables

All baseline data in this study were extracted from the hospital information system. These data include patient age, sex, body mass index (BMI), smoking status (categorized as never or ever smokers), and medical history of chronic diseases such as CAD, hypertension, diabetes, chronic kidney disease, and heart failure. BMI was calculated by dividing body weight (kg) by the square of height (m^2^). CAD was defined as prior myocardial infarction, coronary revascularization, or coronary angiography showing ≥50% stenosis. Hypertension was defined as systolic blood pressure ≥140 mmHg, diastolic blood pressure ≥90 mmHg, current use of antihypertensive medication, or self-reported history of hypertension. Diabetes was defined as fasting plasma glucose ≥126 mg/dL, current use of antidiabetic medication, or self-reported history of diabetes. Chronic kidney disease was defined as an estimated glomerular filtration rate <60 mL/min/1.73 m^2^ persisting for ≥3 months, or the presence of other markers of kidney damage. Heart failure was diagnosed based on the presence of typical symptoms and signs of cardiac pump dysfunction, in conjunction with confirmatory findings from auxiliary examinations (e.g., echocardiography).

Laboratory test results, such as complete blood count, Hemoglobin A1c (HbA1c), total cholesterol (TC), triglycerides (TG), LDL-C, high-density lipoprotein cholesterol (HDL-C), and hs-CRP, were also included in the evaluation. Based on these indicators, we calculated the following derived parameters: RC, RCII, platelet-to-lymphocyte ratio (PLR), neutrophil-to-lymphocyte ratio (NLR), and monocyte-to-lymphocyte ratio (MLR). The specific calculation formulas are as follows: PLR = platelet count/lymphocyte count; NLR = neutrophil count/lymphocyte count.

In addition, coronary angiography findings, including left main disease, multivessel disease, chronic total occlusion, and in-stent restenosis, were considered important reference data.

### Definition of RCII

RC=TC−(HDL−C+LDL−C);RCII=RC(mg/dL)×hs−CRP(mg/L)/10.

### Outcomes

As no deaths occurred in this study, the primary outcome was the incidence of new MACE, including non-fatal myocardial infarction and non-fatal stroke. MACE events were collected through a specially designed questionnaire system and confirmed during the follow-up period. Patients were followed up every six months by trained physicians through telephone consultations and/or outpatient visits. All reported events were verified against electronic medical records at our center or through collection of medical records from other hospitals. Patients who could not be contacted after at least three attempts at a scheduled follow-up point were considered lost to follow-up; those with missing follow-up data were excluded from the analysis (*n* = 1,590). The first occurrence of a MACE event was classified as a “first event,” with the onset time determined by the report date. For participants who experienced multiple events, only the first event was included in the primary outcome analysis. For participants who did not report MACE events, the follow-up time was calculated based on the interval between the baseline assessment and the final survey date.

## Statistical analysis

Baseline characteristics were summarized according to the presence or absence of MACE. The Kolmogorov–Smirnov (KS) test is used to assess the normality of continuous variables. No data transformation was performed; all continuous variables were analyzed in their original scale. Continuous variables with non-normal distribution were presented as median (interquartile range, IQR), while normally distributed continuous variables were presented as mean (standard deviation, SD). Categorical variables were expressed as number (%). Comparisons between continuous variables were performed using the *t*-test or Wilcoxon rank-sum test, and comparisons between categorical variables were performed using the chi-square test. The cumulative incidence of MACE across different RCII groups (categorized by tertiles) was depicted using Kaplan–Meier curves and compared using the log-rank test. The relationship between RCII and MACE was estimated using univariable and multivariable Cox proportional hazards models, with results expressed as hazard ratios (HR) and 95% confidence intervals (CI). The proportional hazards assumption was formally tested using Schoenfeld residuals, and no significant violations were detected (global *P* > 0.05). The univariable model was unadjusted. Variables with *P* < 0.05 in the univariable analysis were subsequently included in the multivariable Cox regression model for adjustment. Multicollinearity among covariates in the multivariable model was assessed using the variance inflation factor (VIF), and all variables had a VIF < 5, indicating no significant multicollinearity. The multivariable model adjusted for age, BMI, history of CAD, hypertension, diabetes, chronic kidney disease, TC, TG, HbA1c, PLR, NLR, multivessel disease, chronic total occlusion, and in-stent restenosis. Subgroup analyses were conducted based on age, sex, smoking status, history of coronary artery disease, diabetes, and hypertension. Covariates in the models were carefully selected based on their association with the outcome, supported by existing scientific knowledge. The potential non-linear relationship between RCII and MACE was further evaluated using restricted cubic spline (RCS) regression. Finally, the predictive ability of receiver operating characteristic (ROC) curves at four time points was assessed for predicting MACE risk. A two-sided *P* < 0.05 was considered to be statistically significant. All analyses were performed with R version 4.3.1 (R Foundation for Statistical Computing, Vienna, Austria).

## Results

### Study population baseline characteristics

A total of 2,171 participants were included in the analysis, with a median follow-up duration of 36 months. The mean age was 65.32 ± 6.85 years, and 1,334 participants (61.4%) were male. Compared to the non-MACE group, participants in the MACE group exhibited significant differences in several baseline characteristics. The MACE group was generally older, had a higher BMI, and a higher prevalence of CAD, hypertension, diabetes, and chronic kidney disease. Additionally, the MACE group had higher levels of TG, TC, LDL-C, HbA1c, RC, RCII, hsCRP, PLR, NLR, and MLR. This group also had a higher proportion of multivessel disease, chronic total occlusion, and in-stent restenosis (Table 1).

**Table 1:** Baseline characteristics of participants according to MACE.

Variable	All participants (*n* = 2,171)	Non-MACE groups (*n* = 1,808)	MACE groups (*n* = 363)	*P* value
Age (years, median ± SD)	65.32 ± 6.85	64.90 ± 7.14	67.44 ± 4.68	<0.001
Males (*n*, %)	1,334 (61.4)	1,105 (61.1)	229 (63.1)	0.520
BMI (kg/m^2^, median, IQR)	24.68 (23.44, 26.14)	24.62 (23.36, 26.14)	24.82 (23.87, 26.07)	0.006
Ever smokers (*n*, %)	495 (22.8)	399 (22.1)	96 (26.4)	0.081
History of CAD (*n*, %)	620 (28.6)	492 (27.2)	128 (35.3)	0.002
Hypertension (*n*, %)	1,353 (62.3)	1,087 (60.1)	266 (73.3)	<0.001
Diabetes (*n*, %)	679 (31.3)	514 (28.4)	165 (45.5)	<0.001
Chronic kidney disease (*n*, %)	103 (4.7)	76 (4.2)	27 (7.4)	0.012
Heart failure (*n*, %)	408 (18.8)	339 (18.8)	69 (19)	0.967
TG (mmol/L, median, IQR)	1.36 (0.97, 1.94)	1.31 (0.95, 1.86)	1.65 (1.08, 2.48)	< 0.001
TC (mmol/L, median, IQR)	4.44 (3.72, 5.19)	4.36 (3.64, 5.12)	4.77 (4.1, 5.52)	< 0.001
LDL-C (mmol/L, median, IQR)	2.66 (2.04, 3.33)	2.62 (2, 3.32)	2.87 (2.31, 3.48)	< 0.001
HDL-C (mmol/L, median, IQR)	1.2 (1.01, 1.4)	1.21 (1.01, 1.4)	1.17 (1, 1.4)	0.422
HbA1c (%, median, IQR)	5.8 (5.6, 6.2)	5.8 (5.6, 6.1)	5.9 (5.6, 6.3)	0.006
RC (mg/dL, median, IQR)	18.17 (11.99, 27.84)	17.79 (11.21, 26.68)	22.43 (15.08, 33.26)	< 0.001
RCII (median, IQR)	3.14 (1.76, 5.75)	2.85 (1.52, 5.17)	4.88 (2.96, 7.98)	< 0.001
hsCRP (mg/L, median, IQR)	1.82 (1.09, 2.76)	1.74 (1.04, 2.64)	2.44 (1.6, 3.27)	< 0.001
PLR (median, IQR)	155.56 (113.64, 215.18)	153.63 (110.87, 212.34)	162.73 (126.52, 234.4)	0.001
NLR (median, IQR)	4.1 (2.54, 6.35)	4 (2.53, 6.14)	4.93 (2.73, 7.6)	< 0.001
MLR (median, IQR)	0.29 (0.21, 0.4)	0.29 (0.21, 0.39)	0.32 (0.23, 0.46)	< 0.001
Left main disease (*n*, %)	114 (5.3)	89 (4.9)	25 (6.9)	0.161
Multivessel disease (*n*, %)	537 (24.7)	425 (23.5)	112 (30.9)	0.004
Chronic total occlusion (*n*, %)	184 (8.5)	136 (7.5)	48 (13.2)	<0.001
In-stent restenosis (*n*, %)	82 (3.8)	59 (3.3)	23 (6.3)	0.008

BMI, body mass index; IQR, interquartile range; CAD, coronary artery disease; TG, triglycerides; TC, total cholesterol; LDL-C, low-density lipoprotein cholesterol; HDL-C, high-density lipoprotein cholesterol; HbA1c, hemoglobin A1c; RC, remnant cholesterol; RCII, remnant cholesterol-inflammation index; hs-CRP, C-reactive protein; PLR, platelet-to-lymphocyte ratio; NLR, neutrophil-to-lymphocyte ratio; MLR, monocyte-to-lymphocyte ratio.

### Association between RCII and MACE

**Figure 2:**
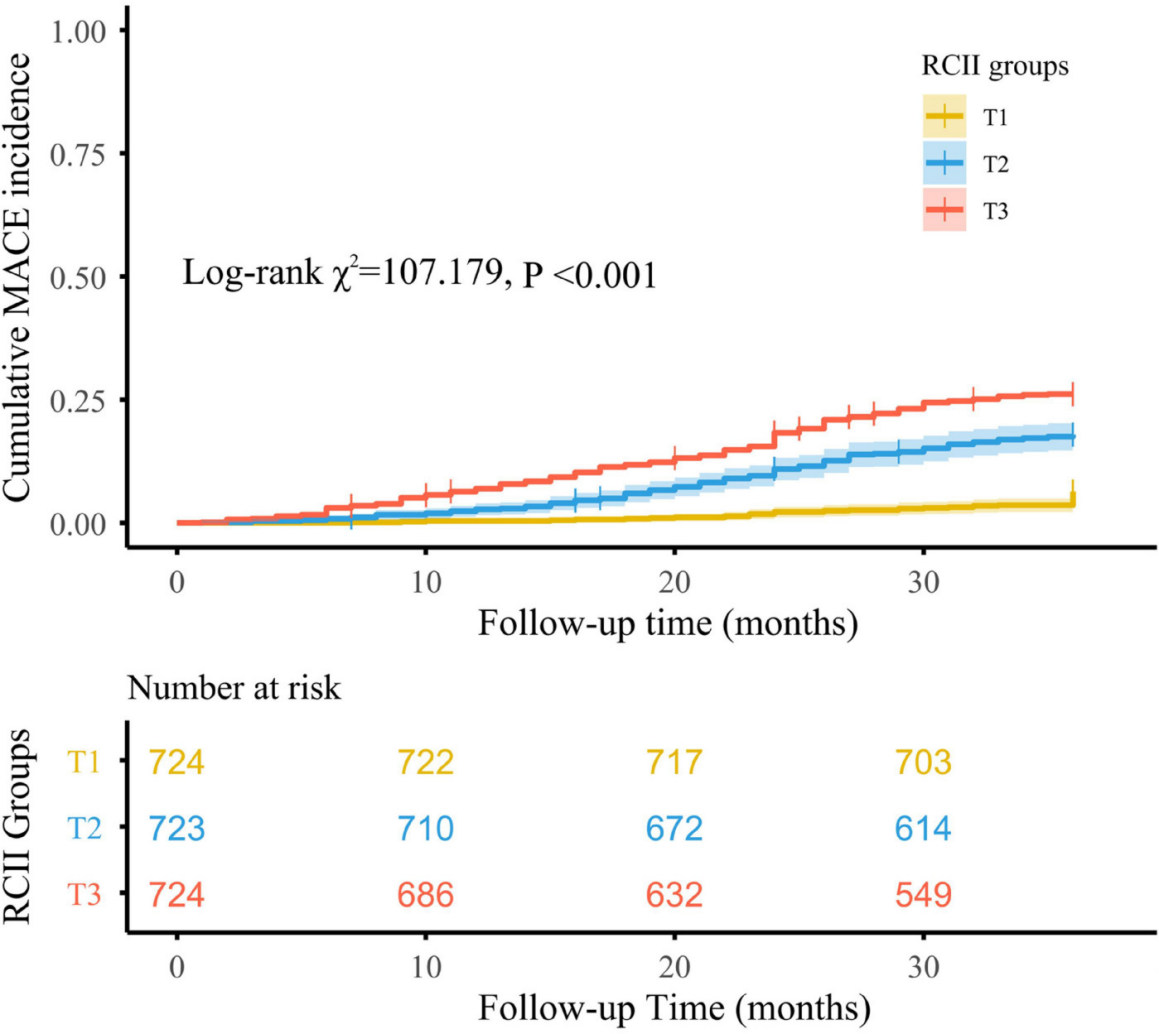
Kaplan–meier curve of cumulative MACE incidence stratified by RCII tertiles. The cutoff values for RCII tertiles were: T1, <2.206; T2, 2.206–4.680; T3, ≥ 4.680. Number at risk is shown below the *x*-axis. MACE, major adverse cardiovascular events; T, tertiles.

During the follow-up period, 363 cases (16.7%) of MACE were observed. As shown in Figure 2, the Kaplan–Meier cumulative incidence curves of MACE, stratified by RCII tertiles, demonstrated significant differences (Log-rank *χ*^2^ = 107.179, *P* < 0.001), indicating a graded increase in MACE risk with higher RCII levels.

Cox proportional hazards regression models were further used to explore the association between RCII and the risk of MACE (Table 2). The univariable model showed that for each 1 SD deviation increase in RCII, the risk of MACE increased by 10% (HR = 1.10, 95%CI: 1.08–1.11). Compared to T1, the HR for RCII in the T2 and T3 tertiles were 3.05 (95%CI: 2.18–4.27) and 4.77 (95%CI: 3.46–6.59), respectively. In the multivariable model, each SD deviation increase in RCII was associated with a 5% increase in MACE risk (HR = 1.05, 95%CI: 1.04–1.07). Compared to T1, the HR for RCII in the T2 and T3 tertiles were 2.87 (95%CI: 2.03–4.04) and 4.42 (95%CI: 3.09–6.32), respectively.

**Table 2:** HR (95% CI) of MACE by RCII in two models.

	Univariable model			Multivariable model		
Variable	HR (95%CI)	*P* value	*P* for trend	HR (95%CI)	*P* value	*P* for trend
RCII			<0.001			<0.001
Continuous	1.10 (1.08–1.11)	<0.001		1.05 (1.04–1.07)	<0.001	
Categorical						
T1	Ref.			Ref.		
T2	3.05 (2.18–4.27)	<0.001		2.87 (2.03–4.04)	<0.001	
T3	4.77 (3.46–6.59)	<0.001		4.42 (3.09–6.32)	<0.001	

HR, hazard ratio; CI, confidence interval; RCII, remnant cholesterol-inflammation index; T, tertiles.

Multivariable model: adjusted for age, BMI, history of CAD, hypertension, diabetes, chronic kidney disease, TC, TG, HbA1c, PLR, NLR, multivessel disease, chronic total occlusion, and in-stent restenosis.

### Subgroup analysis of RCII and MACE

Subgroup analysis revealed that RCII was significantly associated with MACE risk across multiple subgroups (Table 3). Specifically, RCII was associated with an increased risk of MACE in both individuals aged <65 and ≥65 years, as well as in males, females, smokers, and non-smokers. Furthermore, RCII was significantly associated with an increased risk of MACE regardless of the presence of chronic conditions such as a history of CAD, diabetes, or hypertension. No significant interactions were observed between subgroups, indicating that the effect of RCII on MACE risk is consistent across all subgroups.

**Table 3:** RCII and MACE risk according to different subgroups.

Variable	*n*(%)	HR (95%CI)	*P* value	*P* for interaction
Age				0.276
<65 years	951 (43.8)	1.10 (1.05–1.16)	<0.001	
≥65 years	1,220 (56.2)	1.05 (1.03–1.07)	<0.001	
Sex				0.205
Female	837 (38.6)	1.07 (1.04–1.10)	<0.001	
Male	1,334 (61.4)	1.06 (1.04–1.09)	<0.001	
Smoking				0.119
Never	1,674 (77.2)	1.06 (1.04–1.08)	<0.001	
Current/Past	495 (22.8)	1.08 (1.02–1.14)	0.007	
History of CAD				0.102
No	1,551 (71.4)	1.08 (1.05–1.11)	<0.001	
Yes	620 (28.6)	1.03 (1.01–1.06)	0.018	
Diabetes				0.267
No	1,492 (68.7)	1.09 (1.06–1.12)	<0.001	
Yes	679 (31.3)	1.03 (1.01–1.06)	0.002	
Hypertension				0.343
No	818 (37.7)	1.08 (1.02–1.14)	0.011	
Yes	1,353 (62.3)	1.05(1.03–1.07)	<0.001	

HR, hazard ratio; CI, confidence interval.

Adjusted for age, BMI, history of CAD, hypertension, diabetes, chronic kidney disease, TC, TG, HbA1c, PLR, NLR, multivessel disease, chronic total occlusion, and in-stent restenosis.

### Non-linear associations of RCII and MACE

**Figure 3:**
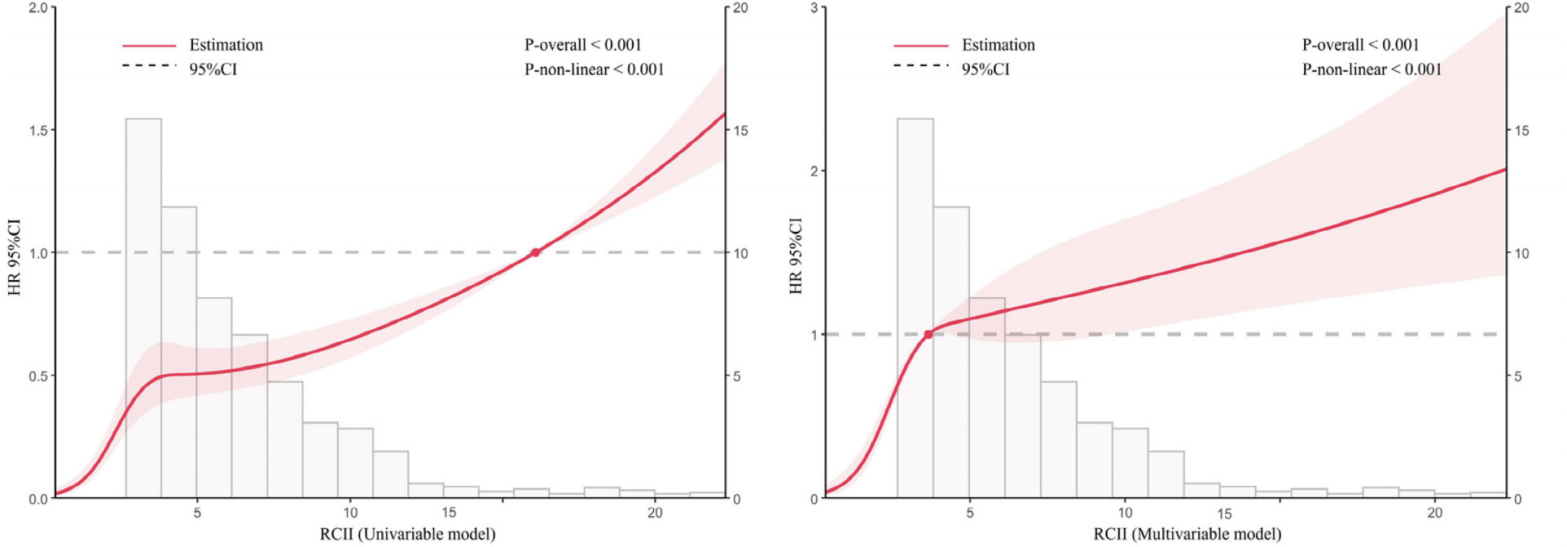
Dose-response association between RCII and MACE. The solid line represents the estimated HR, and the shaded area represents the 95% CI. *P* for non-linearity < 0.001. The risk increased steeply up to an RCII of approximately 2.302 (univariable) and 2.354 (multivariable), after which the slope attenuated, suggesting a threshold effect. HR, hazard ratio; CI, confidence interval; RCII, remnant cholesterol-inflammation index. Multivariable model: adjusted for age, BMI, history of CAD, hypertension, diabetes, chronic kidney disease, TC, TG, HbA1c, PLR, NLR, multivessel disease, chronic total occlusion, and in-stent restenosis.

RCS analysis further demonstrated a non-linear positive association between RCII and MACE (*P* for non-linearity < 0.001, Figure 3). The risk of MACE increased steeply until RCII reached approximately 2.302 in the univariable model and 2.354 in the multivariable-adjusted model, beyond which the risk increase plateaued, suggesting a threshold effect.

### Predictive ability of RCII

**Figure 4:**
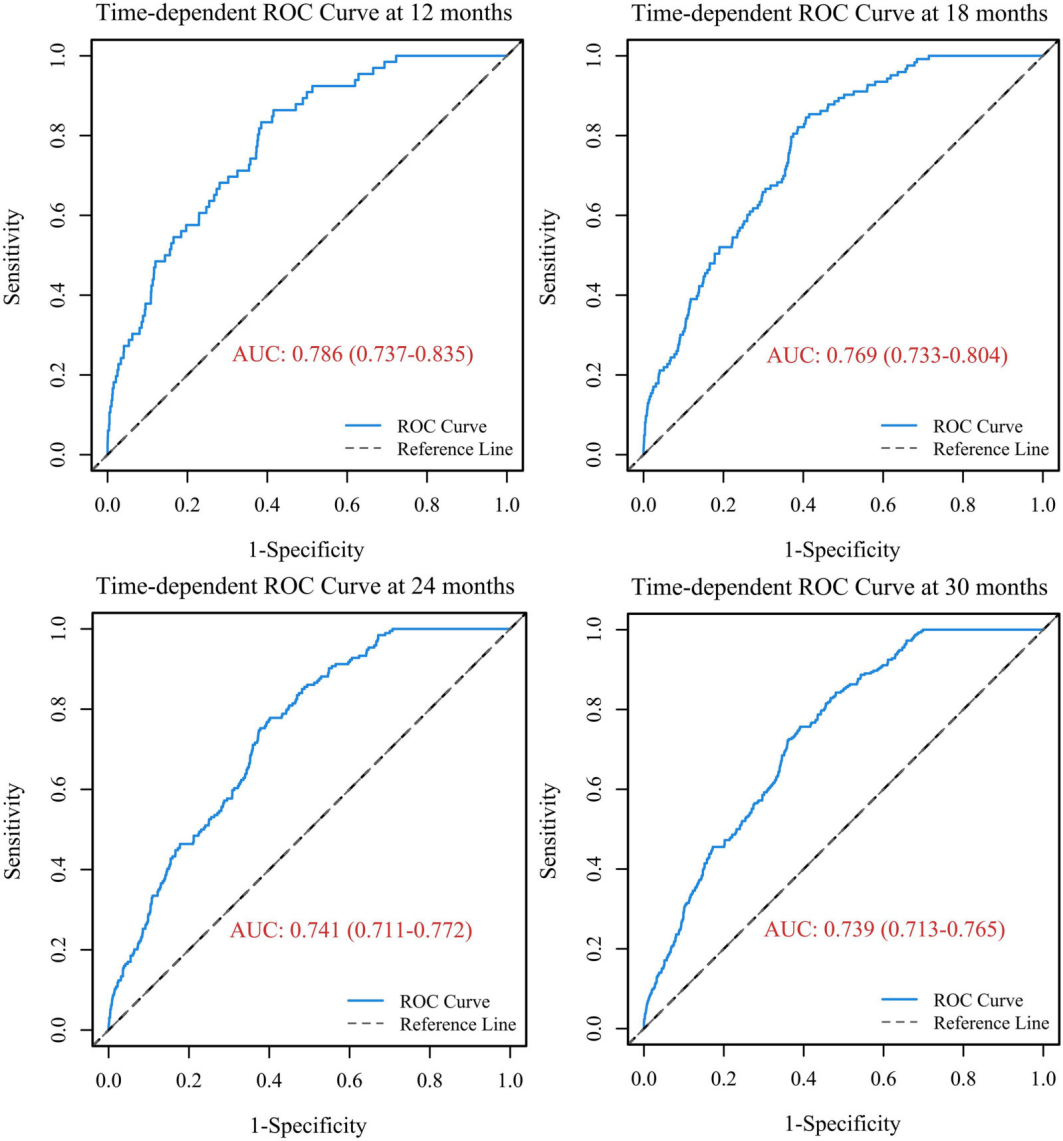
Time-dependent ROC curve for the RCII and MACE. ROC, receiver operating characteristic; AUC, area under the curve.

Time-dependent ROC analysis revealed that RCII demonstrated moderate discriminatory ability in predicting MACE risk (Figure 4). At 12 months, the AUC was 0.786 (95%CI 0.737–0.835), with an optimal cutoff of 2.648, sensitivity of 83.3%, and specificity of 61.5%. At 18 months, the AUC was 0.769 (95%CI 0.733–0.804), with an optimal cutoff of 2.899, sensitivity of 85.4%, and specificity of 58.5%. At 24 months, the AUC was 0.741 (95%CI 0.711–0.772), with an optimal cutoff of 2.899, sensitivity of 77.8%, and specificity of 59.8%. At 30 months, the AUC was 0.739 (95%CI 0.713–0.765), with an optimal cutoff of 2.942, sensitivity of 75.7%, and specificity of 60.9%.

## Discussion

This study is a retrospective cohort investigation aimed at evaluating the association between the RCII and MACE in patients with angina following PCI. We utilized the Cox proportional hazards model to assess the relationship between RCII and MACE risk, adjusting for potential confounding factors such as age, BMI, chronic kidney disease, and diabetes. Our findings demonstrate that RCII remains independently and positively associated with MACE (HR 1.05, 95%CI 1.04–1.07). Further RCS analysis revealed a non-linear relationship between RCII and MACE, with a sharp increase in risk at lower RCII levels. Once RCII surpassed a specific threshold, the risk increase plateaued. Subgroup analyses confirmed that the effect of RCII was consistent across different populations, suggesting that it is a widely applicable prognostic marker in various patient groups.

Atherosclerosis is universally recognized as a chronic inflammatory disease, with its underlying mechanism involving lipid deposition in the vascular endothelium, which subsequently triggers an inflammatory response. RC, defined as the difference between TC and the sum of HDL-C and LDL-C, has emerged as a critical marker of cardiovascular risk. RC is composed of triglyceride-rich remnants, including IDL and VLDL, which are highly atherogenic. These remnants can penetrate the endothelium, promoting lesion progression within the arterial wall (16). A systematic review and meta-analysis by Delialis et al., encompassing 29 studies with a total of 257,387 participants, established a significant association between elevated RC levels and increased risk of atherosclerotic cardiovascular disease, a relationship that holds true in both primary and secondary prevention populations (17). Cao et al. demonstrated that in prediabetic patients with concurrent coronary artery disease, higher RC levels were significantly associated with MACE, suggesting that RC could be an important target for intervention in this specific group with impaired glucose metabolism (18). In addition to lipid metabolism residual risk, inflammatory states play a pivotal role in driving cardiovascular events. A systematic review and meta-analysis by Piccolo et al., encompassing 27 studies with a total of 193,761 participants, identified CRP as an inflammation biomarker independently linked to MACE risk, even after adjusting for traditional risk factors, with a non-linear increase in risk at concentrations between approximately 2 and 4 mg/L (19). A large-scale cohort study showed that when hs-CRP levels were ≥2 mg/L, the MACE risk in patients with atherosclerotic cardiovascular disease increased by approximately 22% (20). The interplay between RC and inflammatory risk in PCI patients has been further characterized in recent studies. In a cohort study involving 1,716 acute coronary syndrome patients who underwent PCI, RC levels above the 75th percentile were strongly linked to a higher risk of MACE during follow-up (21). A systematic review of 13,604 PCI patients demonstrated that persistent high residual inflammatory risk, defined as hs-CRP ≥2 mg/L at 1 month post-PCI, was associated with significantly increased 1-year risks of MACE (RR 1.64) and all-cause mortality (RR 3.25) (22). A large European cohort study of 15,494 statin-treated PCI patients stratified by LDL-C and hs-CRP levels found that the isolated RIR group had the highest MACE incidence (adjusted HR 1.78, 95%CI 1.36–2.33), while the combined residual risk group showed an HR of 1.56 (23).

A retrospective cross-sectional study by Zhang et al. demonstrated a non-linear relationship between the RCII and the severity of coronary artery stenosis, with threshold effect analysis identifying a turning point at 0.64 (24). In this study, we found that RCII is a significant prognostic marker for MACE in patients with angina undergoing PCI. This finding aligns with the broader literature. Regarding stroke risk, both baseline levels and cumulative exposure to higher RCII were significantly associated with an increased risk of stroke, indicating its important contribution to residual cardiovascular risk (25). A longitudinal cohort study using data from the China Health and Retirement Longitudinal Study further indicated that in individuals with chronic kidney disease stages 0–3, higher RCII levels were associated with increased risks of stroke, myocardial infarction, and all-cause mortality (26). Additionally, a large cohort study by Wang et al. showed that elevated RCII levels were significantly linked to an increased risk of all-cause mortality in middle-aged and elderly populations in both the United States and China (27). A retrospective analysis in a Chinese population revealed that elevated RCII was independently associated with poor functional outcomes 90 days after intra-arterial thrombectomy in patients with occlusive stroke of the anterior circulation (28). Despite differences in the populations and specific outcomes, these studies collectively establish a robust association between RCII and cardiovascular disease, as well as its prognosis, thereby reinforcing the reliability of our findings.

The biological mechanisms explaining the prognostic impact of RCII can be further elucidated. Unlike LDL-C, RC-rich remnants can be directly engulfed by macrophages without prior oxidation, leading to foam cell formation and inducing inflammation, thereby accelerating the atherosclerotic process. Additionally, a bidirectional vicious cycle exists between RC and systemic low-grade inflammation: elevated RC promotes inflammation and endothelial activation, which subsequently increases hs-CRP levels, further disrupting lipid metabolism and exacerbating RC elevation (29). This mechanism has been supported by research at various levels: Mendelian randomization studies have confirmed a causal relationship between the two (30, 31), and clinical observations suggest a synergistic effect in increasing the risk of atherosclerotic cardiovascular disease (32). For instance, a Danish observational study found that the co-existence of high RC and high hs-CRP levels was associated with a greater risk of cardiovascular disease and all-cause mortality than either factor alone (33). Similarly, a prospective cohort study in China indicated that elevated RC and hs-CRP levels together posed the highest risk for new-onset stroke, surpassing the risk of either factor alone (34). RCII, by integrating lipid-driven and inflammation-driven risk pathways, likely amplifies these synergistic effects, contributing to vascular injury, metabolic dysregulation, and impaired immune surveillance, which ultimately increase the risk of cardiovascular events. In the post-PCI population specifically, this bidirectional cycle carries particular relevance. RC-mediated foam cell formation promotes ongoing atherogenesis in non-stented segments, while sustained low-grade inflammation may impair endothelial healing at the stent site, potentially contributing to neointimal hyperplasia and in-stent restenosis. Thus, patients with elevated RCII may remain at dual risk for both stent-related and non-stent-related events despite successful revascularization.

From a clinical perspective, RCII may offer a practical tool for post-PCI risk stratification. As a composite index derived from routine lipid panels and hs-CRP, it can be calculated at no additional cost in patients for whom hs-CRP is available. Our time-dependent ROC analysis identified optimal RCII cut-offs of approximately 2.6–2.9 across follow-up time points. Although these thresholds require external validation, they provide an initial reference for identifying patients who may warrant closer monitoring. Whether patients identified as high-risk by RCII derive incremental benefit from more intensive secondary prevention measures remains to be evaluated in prospective studies. Serial RCII measurements during follow-up may also help assess longitudinal risk trajectories, though this application also requires further validation.

This study has several limitations. First, this was a single-center retrospective analysis, and approximately 77.4% of initially screened patients were excluded. A considerable number of eligible patients were excluded because hs-CRP was not part of the routine pre-PCI laboratory tests at our center. This may have introduced selection bias, as the final analytical sample likely overrepresented patients for whom hs-CRP testing was clinically indicated; hence, the applicability of our findings to unselected PCI populations requires further validation. Second, the primary endpoint was restricted to non-fatal myocardial infarction and non-fatal stroke. No deaths were observed in this cohort during the 36-month follow-up, which may be attributable to the exclusion of patients aged over 80 years. As a result, the endpoint is narrower than the traditional three-point MACE definition. Nonetheless, non-fatal myocardial infarction and stroke represent clinically meaningful events that substantially affect patients' quality of life and long-term prognosis. The associations observed in this study should therefore be interpreted as applying specifically to non-fatal atherothrombotic events rather than to fatal outcomes. Third, data on post-discharge medications, including statin intensity, ezetimibe, PCSK9 inhibitors, and antiplatelet therapy duration, were not available. Data on lifestyle factors such as diet and physical activity were also unavailable. These therapies directly influence RC and hs-CRP levels, and their absence represents an important source of unmeasured confounding. In addition, renal function was analyzed only as a binary variable (chronic kidney disease) rather than by eGFR stages, which may have resulted in inadequate adjustment for renal function. Fourth, RCII was indirectly calculated from standard lipid parameters rather than directly measured, which may introduce some degree of misclassification bias. Fifth, the discriminatory performance of RCII was limited, with AUC values across time points ranging from 0.739 to 0.786. This suggests that RCII alone is insufficient as a standalone predictor and should be integrated with other clinical variables for risk assessment. In addition, formal comparisons of predictive performance between RCII and conventional markers such as LDL-C or hs-CRP using net reclassification improvement and integrated discrimination improvement were not performed; such analyses would help quantify the incremental prognostic value of RCII. Finally, RCII was assessed only at a single baseline time point without longitudinal monitoring, precluding evaluation of its dynamic changes in relation to prognosis. In addition, this study was conducted in a single center in northern China, and the generalizability of our findings to other ethnic or geographic populations remains to be established. Future studies should adopt multicenter prospective designs encompassing diverse populations, incorporating serial RCII measurements and comprehensive medication and lifestyle data to address these limitations.

## Conclusion

In conclusion, our study suggests that baseline RCII is independently associated with the risk of new-onset MACE in patients with angina undergoing PCI, with a graded increase in risk across increasing RCII levels. RCII may therefore represent a potential biomarker for identifying high-risk individuals in clinical practice.

## Data Availability

The raw data supporting the conclusions of this article will be made available by the authors, without undue reservation.
